# Supplementary material for: A prudent planetary limit for geologic carbon storage
Source: Nature. 2025 Sep 3;645(8079):124–32. doi: 10.1038/s41586-025-09423-y (PMC12408384; doi:10.1038/s41586-025-09423-y)
Supplement: Supplementary file 1 — This file contains Supplementary Figs. 1–13. [file 41586_2025_9423_MOESM1_ESM.docx]

Supplemental Information for *A prudent planetary limit for geologic carbon storage*

**Authors:** Matthew J. Gidden^1,2,*^, Siddharth Joshi^1^, John J. Armitage^3^, Alina-Berenice Christ^3^, Miranda Boettcher^4,5^, Elina Brutschin^1^, Alexandre C. Köberle^6,7^, Keywan Riahi^1^, Hans Joachim Schellnhuber^1^, Carl-Friedrich Schleussner^1,8^, Joeri Rogelj^1,9^

**Affiliations:**

^1^ International Institute for Applied Systems Analysis, Laxenburg, Austria

^2^ Center for Global Sustainability, University of Maryland, College Park, USA

^3^ IFP Energies nouvelles, Rueil-Malmaison, France

^4^ German Institute for International & Security Affairs (SWP), Berlin, Germany

^5^ Copernicus Institute of Sustainable Development, Utrecht University, Utrecht, the Netherlands

^6^  Instituto Dom Luiz (IDL), Faculdade de Ciências, Universidade de Lisboa, Lisboa, Portugal

^7^ Potsdam Institute for Climate Impact Research, Potsdam, Germany

^8^ Geography Department and IRITHESys Institute, Humboldt-Universität zu Berlin, Berlin, Germany

^9^ Centre for Environmental Policy and Grantham Institute – Climate Change and Environment, Imperial College London, London, UK

* Corresponding author: gidden@umd.edu


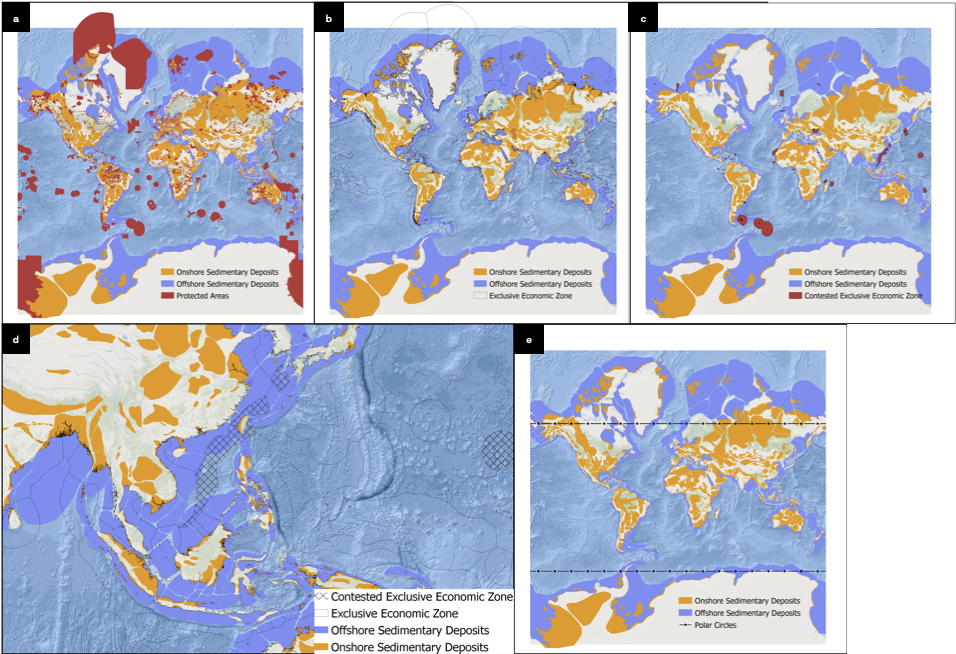


### Fig S1. **a,** visual depiction of protected areas overlayed over offshore and onshore sedimentary deposits. **b,** visual depiction of global EEZ boundaries. **c,** visual depiction of global contested maritime exclusive economic zones, with zoomed in view of South China Sea, **d**. **e**, Visual depiction of polar circles at a global level. We assume that the sedimentary deposits north of Arctic circle and south of Antarctic circle are excluded from the analysis.

Fig S2. Visual depiction of sedimentary deposits excluded from analysis due to seismic hazard caused to PGA >0.115g (Moderate intensity), **a** and >0.401g (Severe intensity), **b**. **c,** Visual depiction of overlay of both the seismic hazard zones on the offshore and onshore sedimentary deposit basins in South America.


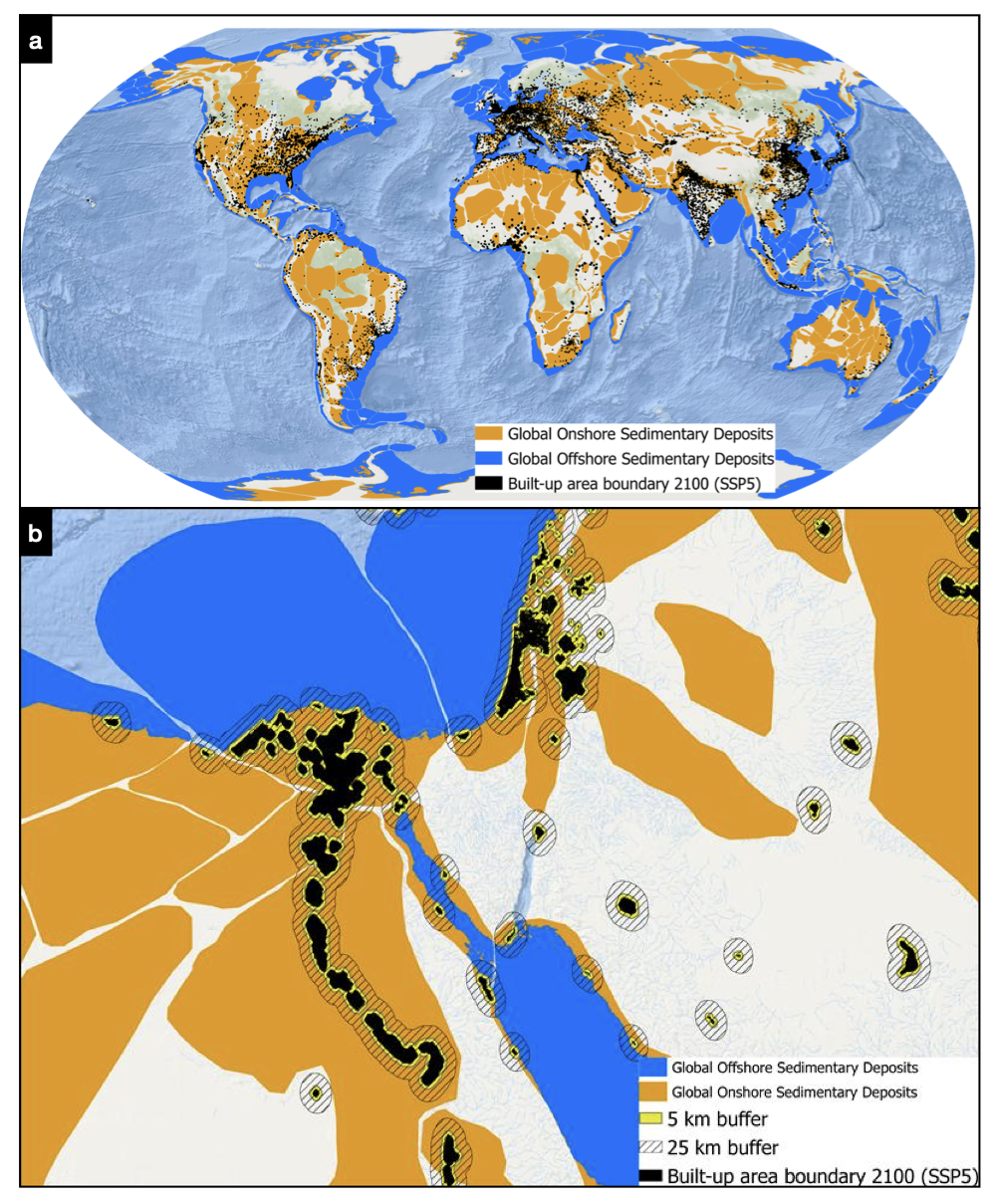
Fig S3. **a**, Visual depiction of built-up areas in 2100 for under SSP5 growth narrative. **b,** visual depiction of overlay of 5km, 25km buffers, and 2100 urban boundaries over portions of Egypt and the Levant.

###

Fig S4. **a**, global map of offshore sedimentary deposit boundaries and water depth. **b,** global map of offshore sedimentary deposit boundaries and offshore oil and gas explorations (blue). **c**, zoomed in view overlaying offshore and onshore oil and gas infrastructure locations in the North Sea on top of 1500m and 300m water depth assessment boundaries. **d**, histogram of count of offshore oil and gas installations for discrete underlying water depth (n=121,717).

Fig S5. **a**, global map of offshore and onshore sedimentary deposit depths. The histograms on the right are depicting the distribution of onshore and offshore sedimentary depths for a 1 decimal degree global grid. **b,** range of calculations for different combinations of minimum and maximum depth values for onshore sedimentary basin for our central case. The vertical line is depicting the maximum assessed injection depth based on literature survey. **c**, heatmap of representative values of assessed geological potential in Gt CO_2_ for a combination of minimum and maximum depth values for onshore sedimentary basin for our central case. The black colored box is representative of our central case and the yellow-colored box is representative of minimum and maximum injection depth based on literature survey.


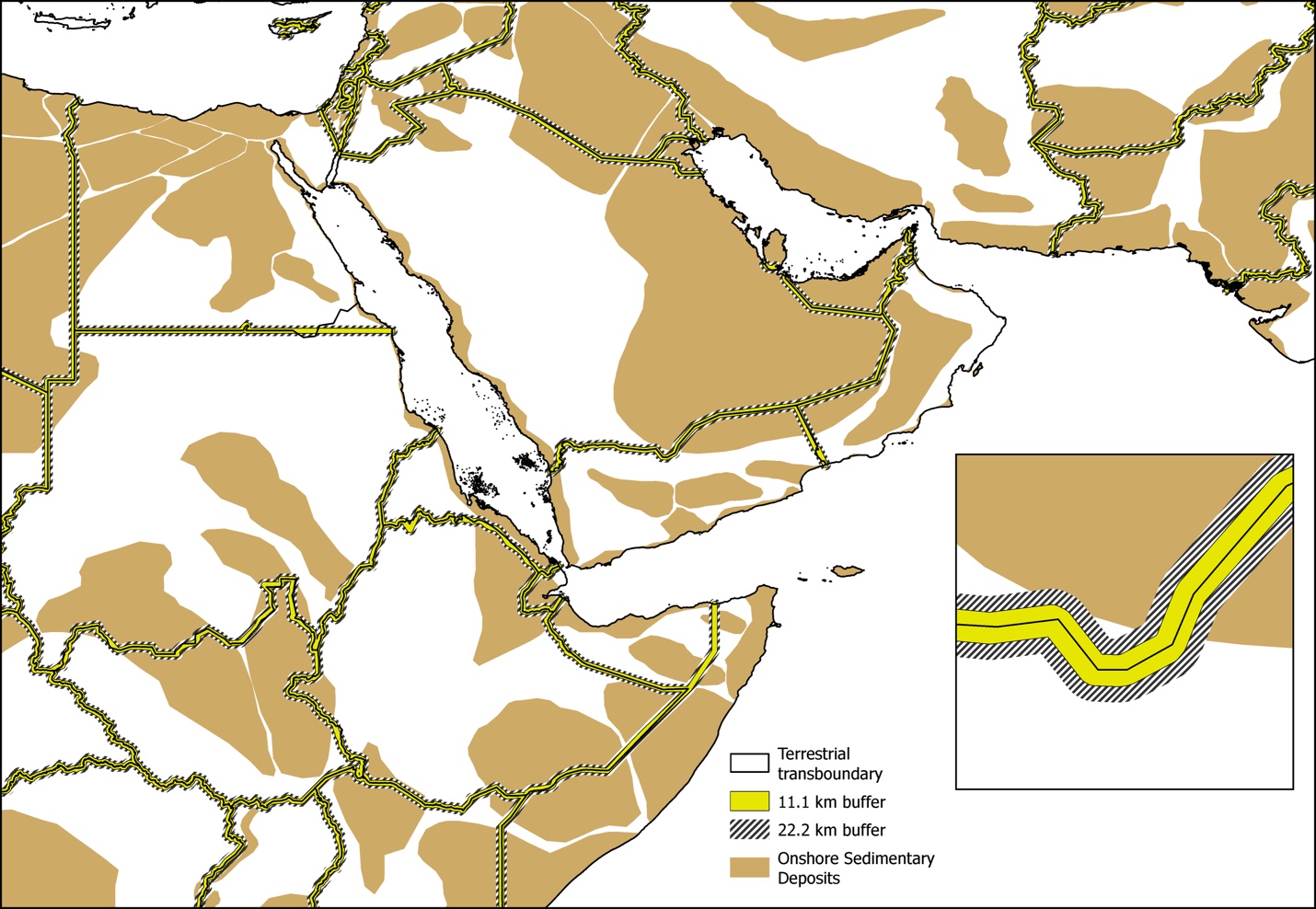
Fig S6. Visual depiction of terrestrial transboundary buffers. The inset figure is a zoomed in view of the buffers on a boundary between Yemen and Saudi Arabia.


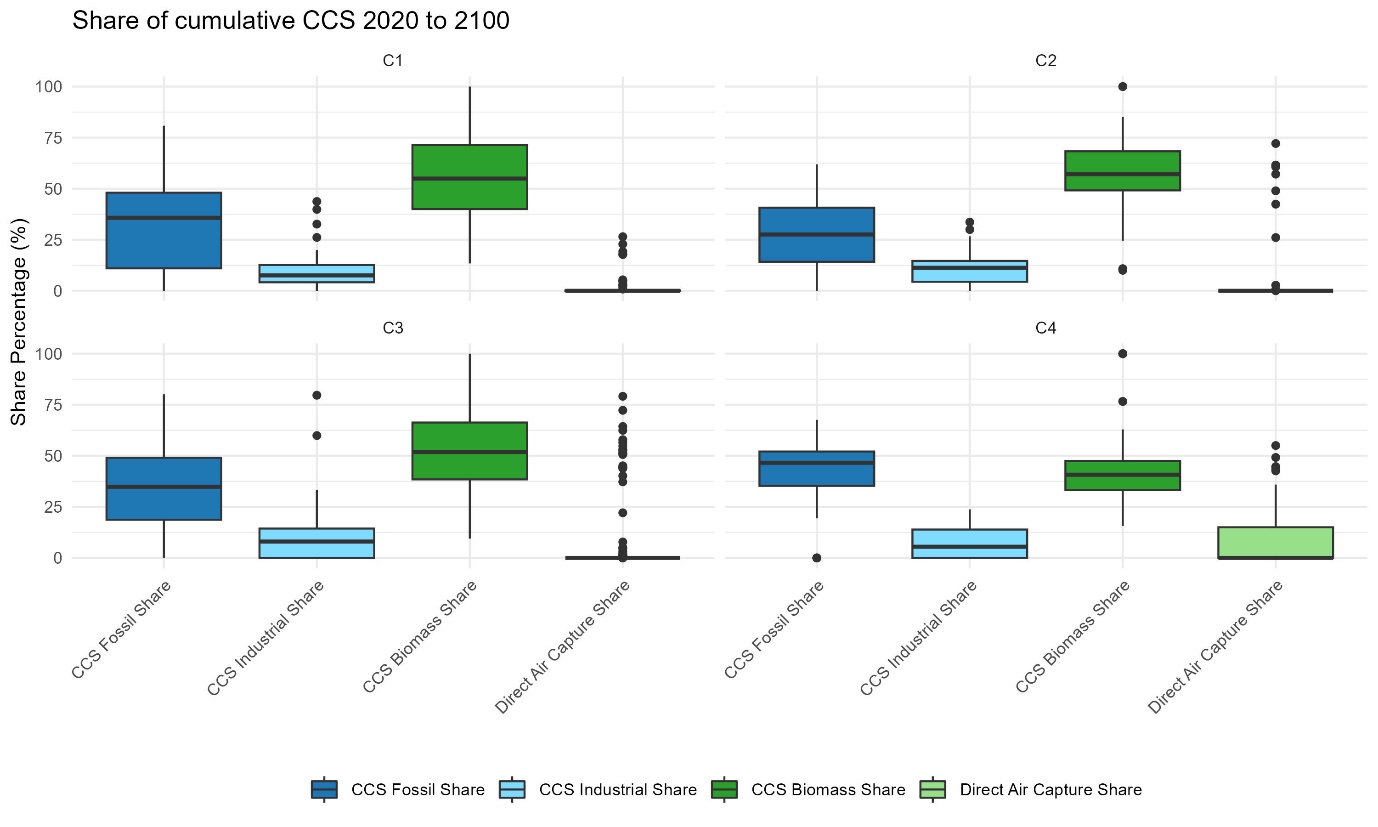


Fig S7. Distribution of carbon sequestration technologies by type, shown as share of cumulative storage (2020-2100). Boxplots illustrate the variation across scenarios, highlighting the relative contributions of fossil CCS, industrial CCS, BECCS, and DACCS. Data are drawn from an unstructured scenario ensemble in the AR6 database, including only scenarios reporting carbon sequestration variables for at least fossil fuels, biomass and industrial processes.


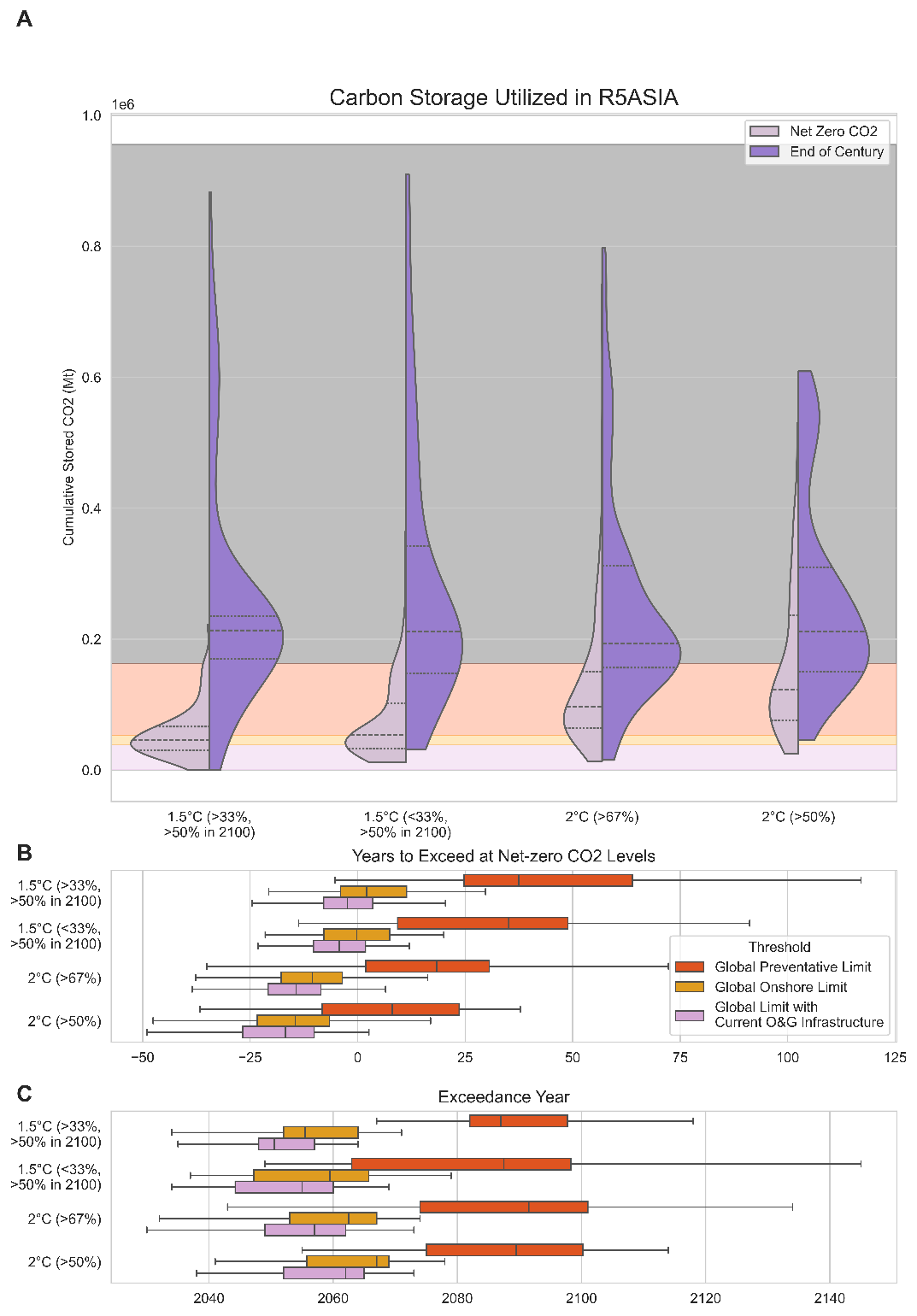


Fig S8. Cumulative carbon storage used in scenarios in the R5ASIA region at net-zero CO_2_ emissions and at the end of the century, **a**. The time at which each regional threshold would be exceeded if yearly carbon storage is maintained at levels when net-zero CO_2_ emissions is reached is shown, **b**, and the year in which each threshold would be exceeded if carbon storage values continue at the same pace after the end of the model horizon, **c**.


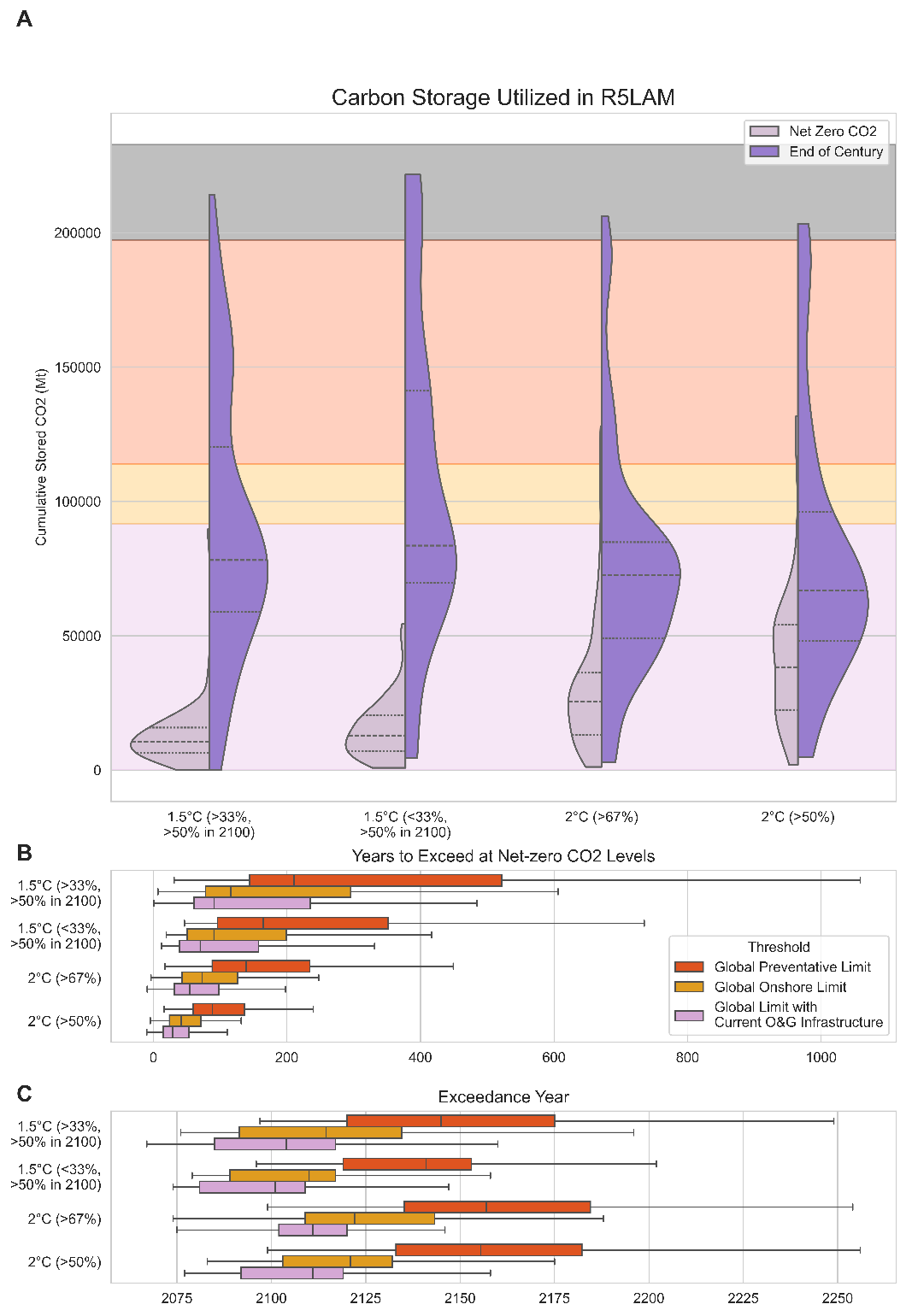


Fig S9. Cumulative carbon storage used in scenarios in the R5LAM region at net-zero CO_2_ emissions and at the end of the century, **a**. The time at which each regional threshold would be exceeded if yearly carbon storage is maintained at levels when net-zero CO_2_ emissions is reached is shown, **b**, and the year in which each threshold would be exceeded if carbon storage values continue at the same pace after the end of the model horizon, **c**.


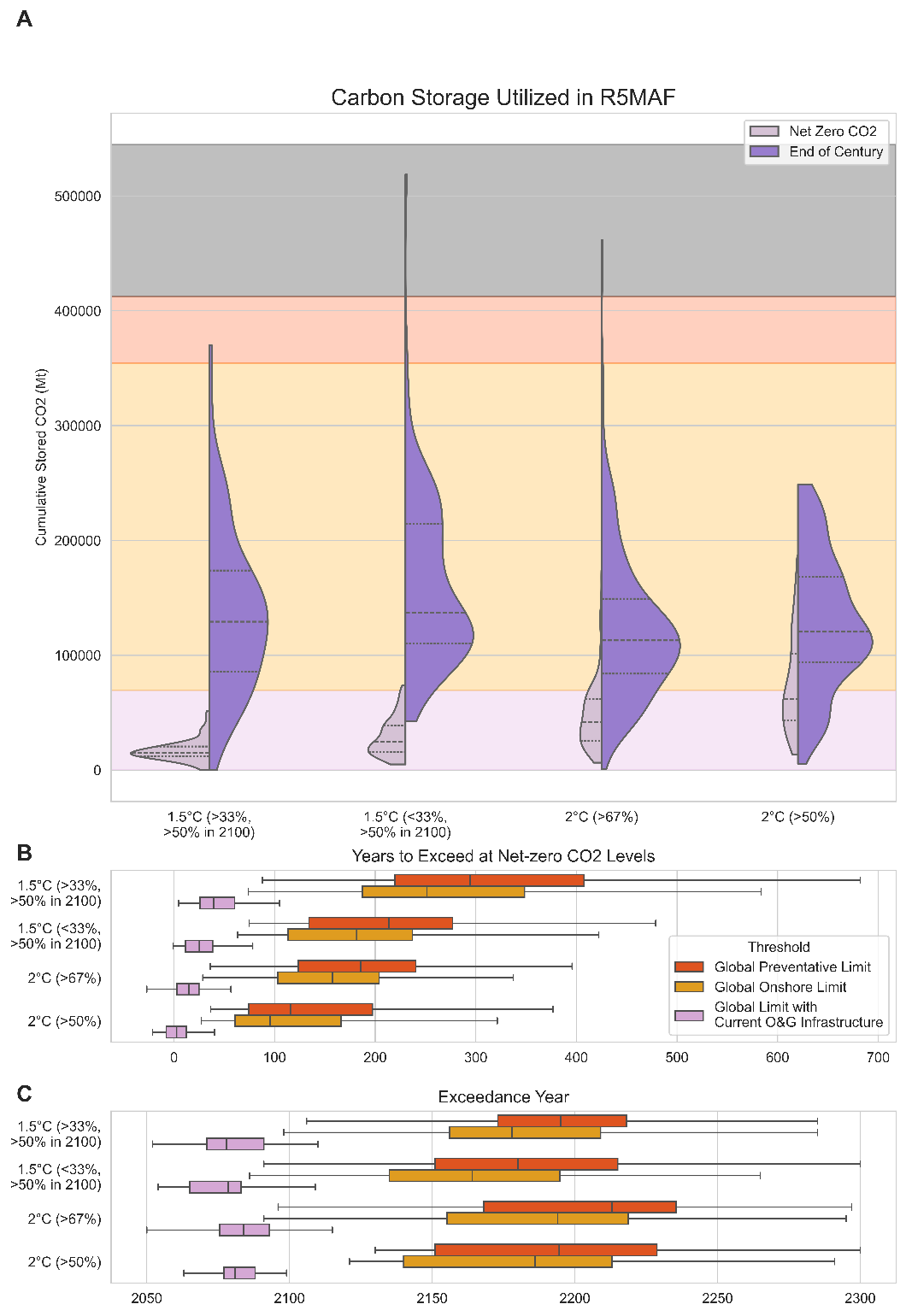


Fig S10. Cumulative carbon storage used in scenarios in the R5MAF region at net-zero CO_2_ emissions and at the end of the century, **a**. The time at which each regional threshold would be exceeded if yearly carbon storage is maintained at levels when net-zero CO_2_ emissions is reached is shown, **b**, and the year in which each threshold would be exceeded if carbon storage values continue at the same pace after the end of the model horizon, **c**.


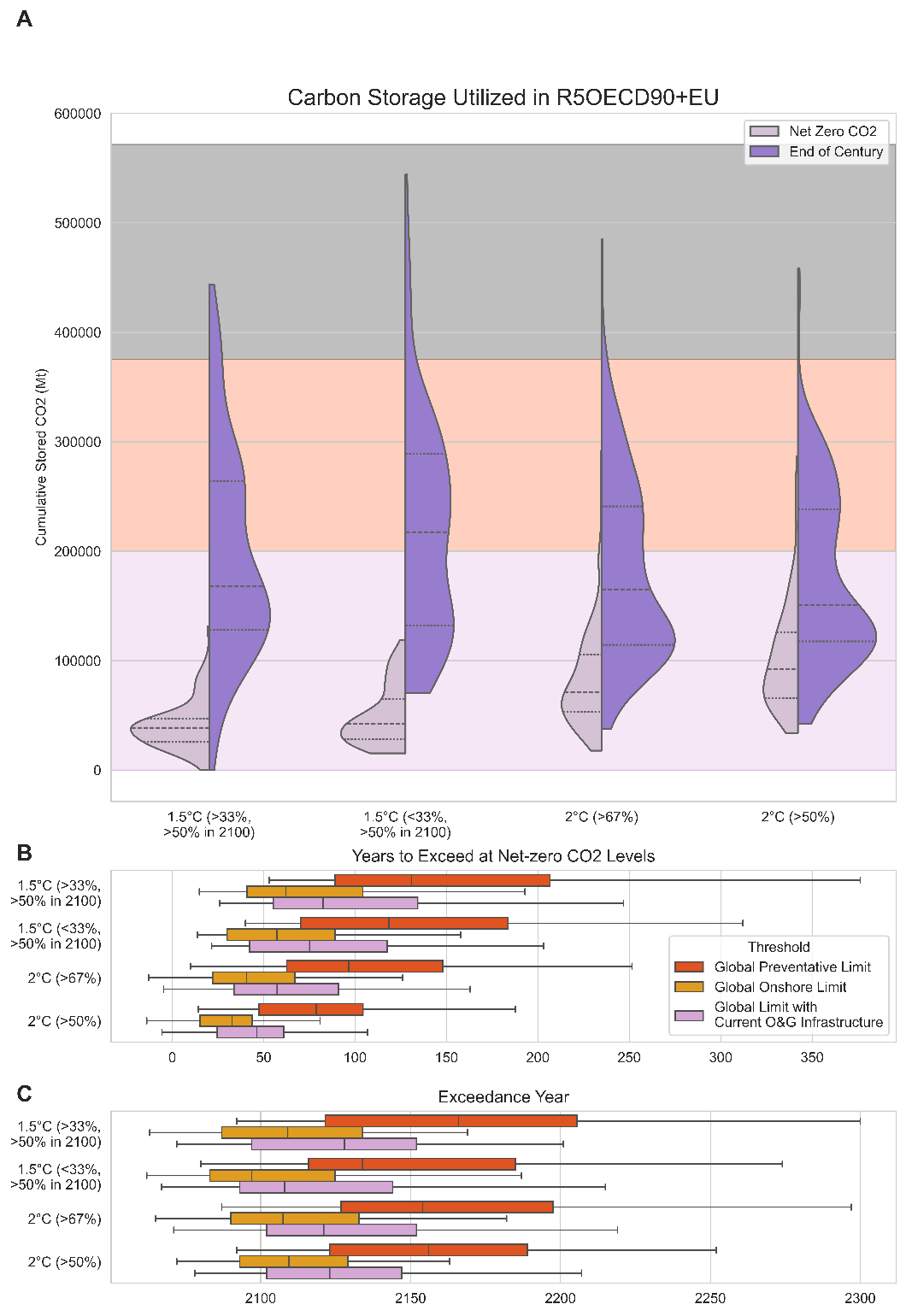


Fig S11. Cumulative carbon storage used in scenarios in the R5OECD+EU region at net-zero CO_2_ emissions and at the end of the century, **a**. The time at which each regional threshold would be exceeded if yearly carbon storage is maintained at levels when net-zero CO_2_ emissions is reached is shown, **b**, and the year in which each threshold would be exceeded if carbon storage values continue at the same pace after the end of the model horizon, **c**.


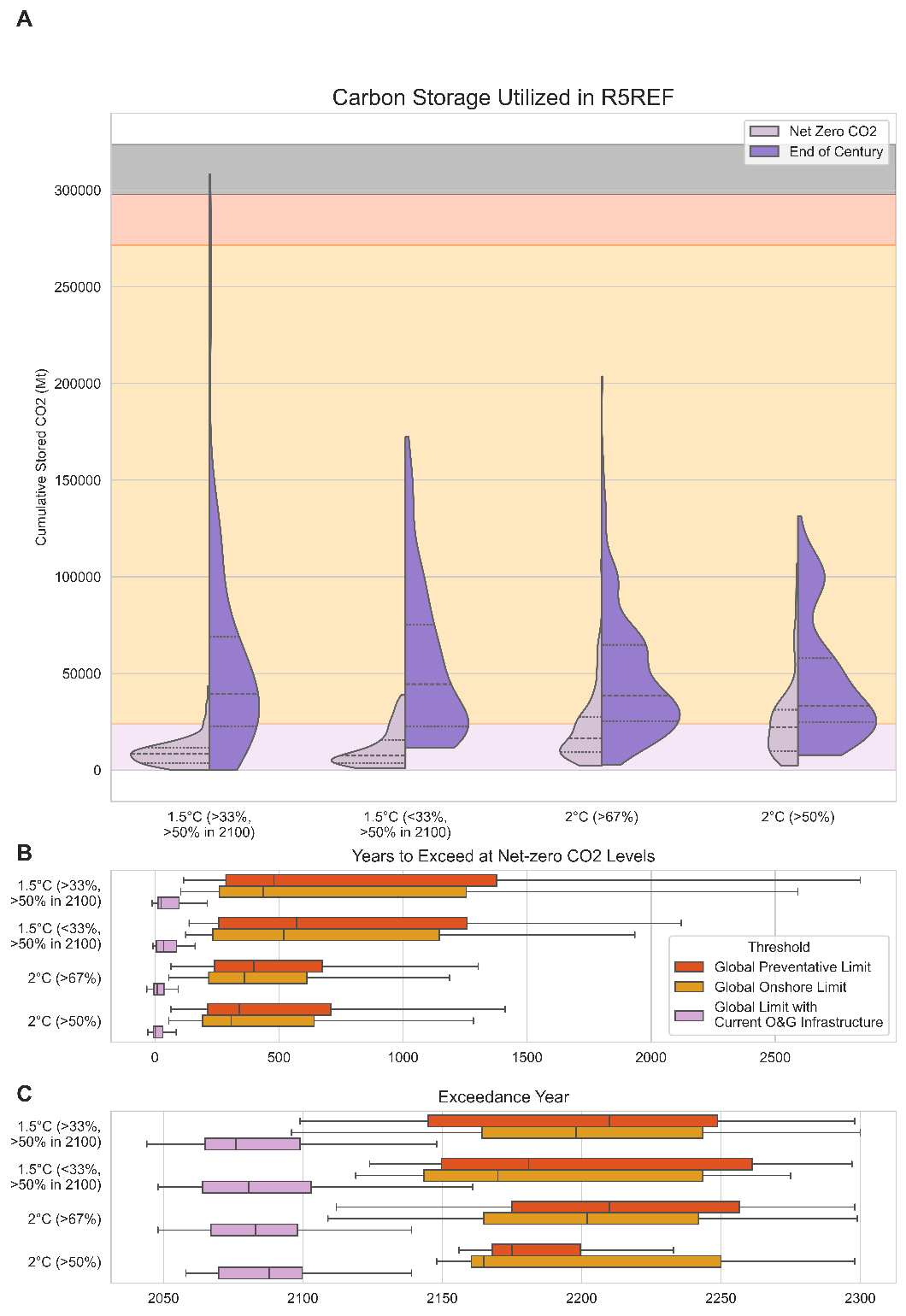


Fig S12. Cumulative carbon storage used in scenarios in the R5REF region at net-zero CO_2_ emissions and at the end of the century, **a**. The time at which each regional threshold would be exceeded if yearly carbon storage is maintained at levels when net-zero CO_2_ emissions is reached is shown, **b**, and the year in which each threshold would be exceeded if carbon storage values continue at the same pace after the end of the model horizon, **c**.


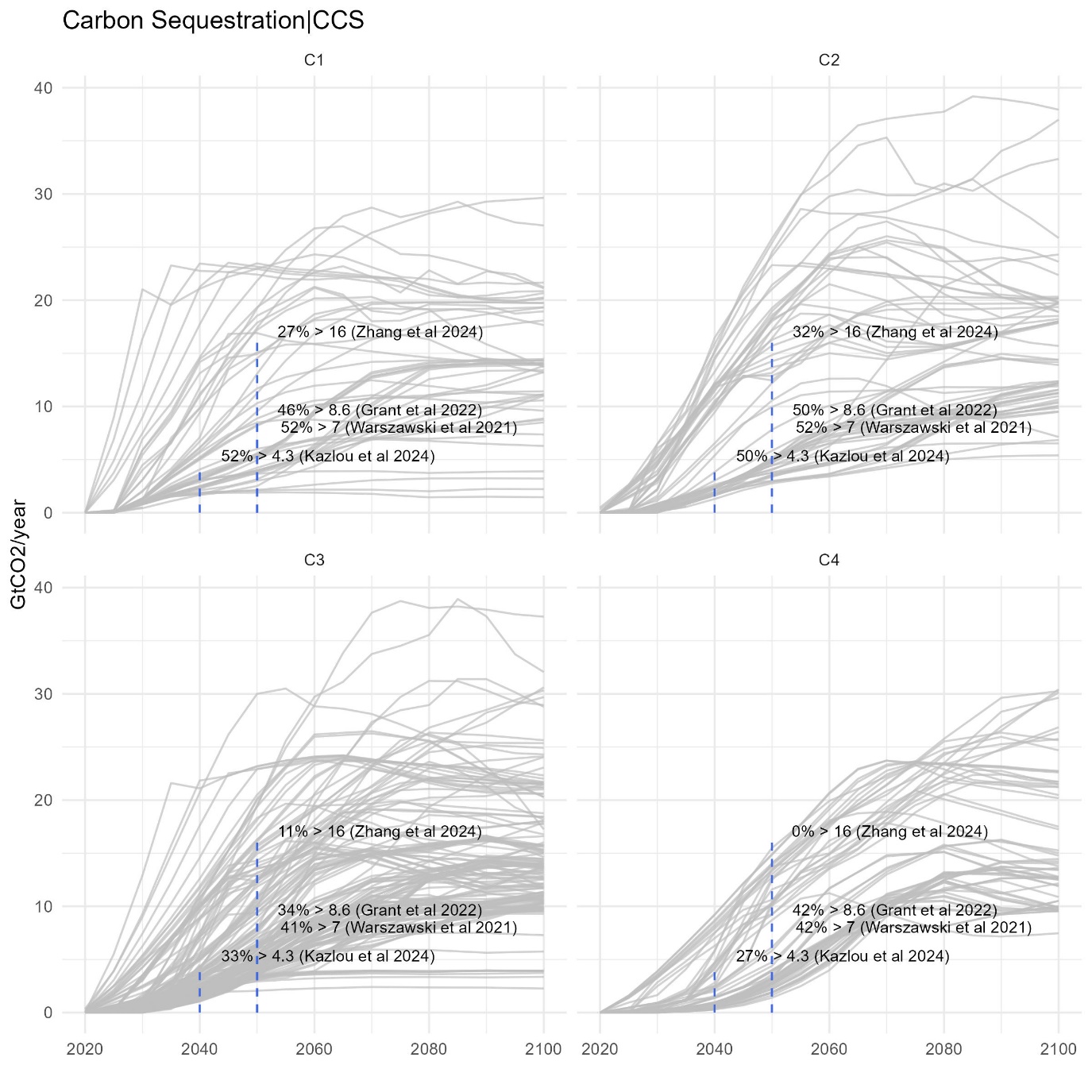


Fig S13. Upscaling trajectories of geologic carbon storage across scenarios over time. Data are drawn from an unstructured scenario ensemble in the AR6 database, including only scenarios reporting carbon sequestration variables for fossil fuels, biomass, and industrial processes. Dashed lines represent optimistic upper bounds from the literature (Grant et al., 2022; Kazlou et al., 2024; Warszawski et al., 2021; Zhang et al., 2024), with percentages indicating the proportion of scenarios exceeding each threshold by category and for a given year. Thresholds overview is based on work by Candelaria Bergero (YSSP Report, 2024). We note that the literature is not monolithic regarding the recognition of CCS growth and injectivity limits as a key constraining criterion for carbon storage in deep mitigation futures. In particular, Ringrose and Meckel (2019) state that “it is clear that the required well rate for realizing global CCS in the 2020–2050 timeframe is a manageable fraction of the historical well rate”.

# Tables

All data tables are provided in a separate file.

Table S1. Global storage volumes considering each geospatial risk consideration layer sequentially, starting from the total global technical potential of onshore storage, offshore storage, and both combined as the global total. Summing all exclusions together results in a global planetary limit, provided at the bottom of the table. An overview of the rationale and quantifications of each limit is provided for every risk consideration. Sensitivity values are provided which estimate the difference in storage based on different assumptions compared to our main estimate for each exclusion layer relative to the previous layer in the main analysis. Exclusion layer sensitivities are described in Table S2.

Table S2. Key sensitivities applied to different exclusion layers. Each sensitivity is labeled based on its Risk Consideration, aligning with tabulated values in Table S1. Negative sensitivities result in lower estimates than the central estimates while positive sensitivities result in higher values. Where sensitivities can be binary (included or not), Y (yes) means they have been included and N (no) means they have not been included. Otherwise, numerical values are provided.

Table S3. A review of available literature which estimates either maximum injection depth, minimum injection depth, or estimates both values. Numerical values are harmonized across sources to provide consistent estimates in meters (m). The majority of the literature we assessed finds that maximal storage depth ranges from around 800-3000m. One study claimed storage depth possible in the gulf of Mexico up to 3500m, but noted that it was unclear about this range due to either pressure in the geopressure zone equilibrating with fracture pressure or loss of permeability. The maximum storage depth we could find was from the USGS which uses a boundary of 3962m based on compression requirements. Taken together, and given the large preponderance of the scientific literature, we maintain a central estimate for maximum storage depth of 2500m, but apply a range between 800m and 3500m in our primary analysis to acknowledge and show the uncertainty in this key parameter in our reported results.

Table S4. A review of countries that currently have explicit policies restricting CCS. Expert judgement is used to estimate whether such policies imply major or minor restrictions and to what degree those policies are subject to change.

Table S5. Country-resolved estimates of onshore, offshore, and total carbon storage are provided for: (1) total technical potential (i.e., without any exclusion layers applied), (2) applying all exclusion layers described in Table S1, and (3) prudent storage estimates including only basins with existing oil and gas infrastructure (i.e., in which storage properties of some part of the basin have already been assessed). We additionally provide the IPCC region in which each country is considered. Countries that do not map to IPCC region categories are included at the bottom of the list. The sum of storage potential across all countries results in the planetary limit.

Table S6. IPCC Scenario categories for the scenarios assessed in this analysis. The IPCC uses shorthand labels (e.g., C1, C2, etc.), while we use the temperature outcomes.

Table S7. A mapping table showing which countries are allocated to each IPCC macro region (so called R5 regions).
